# Columnar grown copper films on polyimides strained beyond 100%

**DOI:** 10.1038/srep13791

**Published:** 2015-09-04

**Authors:** Jeong-Yun Sun, Hae-Ryung Lee, Kyu Hwan Oh

**Affiliations:** 1 Department of Materials Science and Engineering, Seoul National University, Seoul, South Korea, 151-744; 2 Research Institute of Advanced Materials (RIAM), Seoul National University, Seoul, South Korea, 151-744

## Abstract

Many flexible electronic devices contain metal films on polymer substrates to satisfy requirements for both electrical conductivity and mechanical durability. Despite numerous trials to date, the stretchability of metal interconnects remains an issue. In this paper, we have demonstrated a stretchable metal interconnect through control of the texture of a copper film with columnar grown grains on a polyimide (PI) substrate. The columnar grown copper films (CGC films) were deposited by regulating radio frequency (RF) sputtering powers. CGC films were able to sustain their electrical conductivity at strains above 100%. Instead of ultimate electrical discontinuity by channel crack propagation, CGC films maintained their conductivity by forming ligament structures, or a ‘conductive net,’ through trapped micro-cracks. XRD, AFM and *in situ* SEM analysis were used to investigate these stretchable conductors.

Flexible electronics are an extremely popular area of technology, as they to exceed spatial limitations of traditional electronics and can interface directly with living organisms. Many applications, including electronic skin or sensors^1^,^2^, wearable devices^3^, transparent speakers^4^, and flexible and foldable displays^5^,^6^, commonly contain stretchable conductors. Conductive interconnects need to satisfy requirements for both electrical conductivity and mechanical properties. Metals such as gold and copper are preferred as interconnects because of their outstanding electrical properties, but their stretchability has been recognized as a weak point. Many geometrical approaches aim to extend the strain limitations of metals by designing wavy serpentines^7^,^8^,^9^,^10^,^11^,^12^ or facilitating the percolation of conductive particles^13^. In both cases, large stretchability has been demonstrated under uniaxial or multi-axial strain^14^. However, wavy serpentines need extra space near the interconnects to achieve their stretchability^9^, and conductive particles such as silver nanowires show steep increase in resistivity at high strains due to the loss of conductive pathways^15^.

In addition to geometrical approaches, some metallurgical strategies have been studied to enhance the stretchability of conductors. One of these studies demonstrated improved stretchability in copper by annealing and inserting an adhesive layer between a copper film and a polymer substrate^16^. Less commonly, microstructural approaches have been tried, though failure tends to occur due to stress concentrations. Only research on gold nanocrystals on a PDMS substrate^17^,^18^ have been reported, where gold nanocrystals on PDMS substrates endured uniaxial tension up to 30% strain without electrical failures, even as micro-cracks in the gold ligaments developed.

Here, we propose another metallurgical strategy to achieve high stretchability together with high electrical conductivity. We have developed a stretchable copper thin film on a polyimide substrate by controlling its texture. Copper grains were grown in columnar shapes with a particular orientation. The selective grain growth was controlled by changing the radio frequency (RF) sputtering powers. The texture-controlled copper grains were grown in regular arrays with homogeneous diameters and heights above 1 μm, leading to a high aspect ratio. The texture-controlled copper film was stretchable beyond 100% while maintaining its electrical conductivity. Texture-controllable grains were experimentally observable, and their grain growth mechanism is described with crystallographic theories.

## Results and Discussion

**Figure 1:**
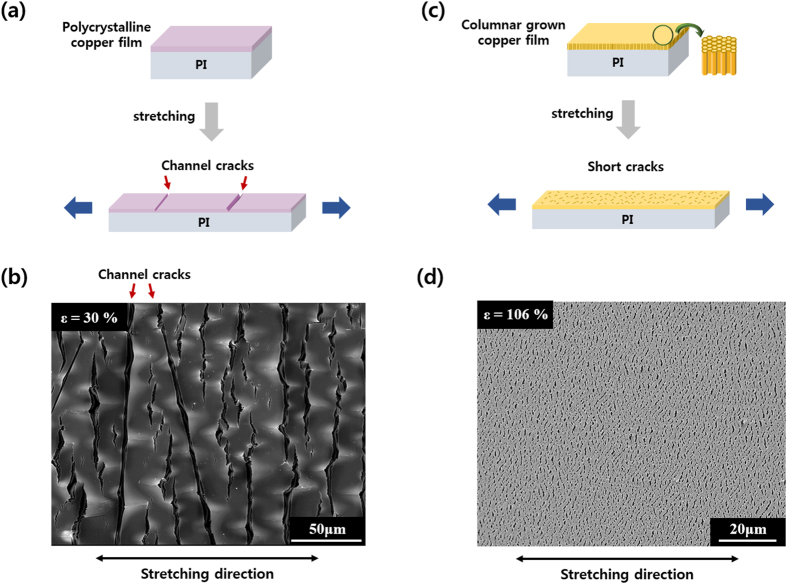
Schematic illustrations and SEM images of sputtered copper films on polyimide (PI) substrates. (**a**) When polycrystalline copper films were subjected to uniaxial tension, they tended to fail by channel cracks, resulting in electrical discontinuity at relatively small strains. (**b**) A scanning electron microscopy (SEM) image of a stretched polycrystalline copper film. A channel crack cut through the film below 10% applied strain. The sample was stretched further up to 30% to clearly show its failure mechanism. Red arrows indicate channel cracks of the polycrystalline film. (**c**) By controlling the texture of copper films, columnar grown copper films (CGC films) could be deposited on PI substrates. When the CGC film was stretched, it developed several short cracks but maintained its electrical conductivity due to ligament connections. (**d**) The CGC film at 106% applied strain under SEM. The CGC film remained interconnected by ligaments with a ‘conductive net’ structure, containing many short-range cracks. Further straining was terminated by the rupture of the PI substrate.

Copper films deposited on polyimide substrates can have different grain growths by varying the radio frequency (RF) sputtering power, e.g., a polycrystalline copper film (Fig. 1a,b) and a columnar grown copper film (CGC film) (Fig. 1c,d). When the polycrystalline film was pulled in uniaxial tension, as shown in Fig. 1, the film failed by channel crack propagation below 10% strain. Buckling in the transverse direction also accompanied deformation, which resulted in delamination and electrical failures in the polycrystalline copper (Fig. 1b). When CGC films were stretched, initial cracks appeared at a strain of 3%; however, these cracks were trapped by surrounding grains instead of propagating, leading to the activation of other cracks. As a result, many short-range cracks developed at low strains, and the microstructure preventing the further development of channel cracks with increasing strain. These trapped short-range cracks contributed to building a ligament structure, such that the films could act as a ‘conductive net’; therefore, the CGC film could maintain its electrical conductivity even at larger strains. In Fig. 1d, the CGC film at 106% applied strain is wholly interconnected by netlike ligaments containing short range-cracks. After 106% strain, further deformation was terminated by the rupture of the PI substrate, not by limitations of the CGC film.

**Figure 2:**
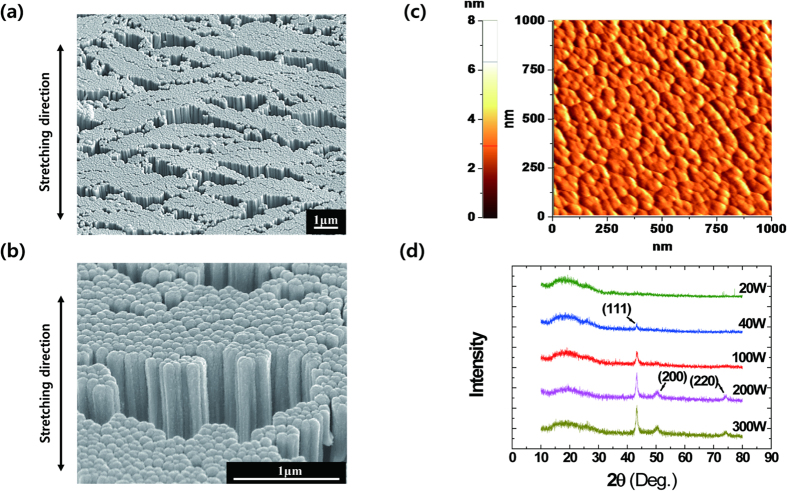
SEM images of CGC films at tilted view angle and data of Cu grain growth. (**a**,**b**) SEM images of stretched CGC thin film at tilt angle of 52°. The CGC film was grown at a sputtering power of 100 W for 60 minutes. Well-aligned Cu columns with nearly uniform height and grains longer than 1 μm were observed. (**c**) AFM data of the CGC film deposited at 100 W for 15 minutes. The average grain size was 43.5 nm and the root mean square (RMS) roughness of the film was 0.829 nm. (**d**) XRD results of sputtered copper thin films with various sputtering powers. For the film sputtered at 20 W, amorphous copper was formed. Columnar grains (111) textures were formed at 40 W and 100 W. At 200 W and 300 W, polycrystalline films of multiple orientations, including (111), (200), (220), were obtained.

A CGC film on a PI substrate deposited at 100 W for 60 minutes was stretched up to 100% and tilted at 52° to examine its grain growth using scanning electron microscopy (SEM) (Fig. 2a,b). The texture-controlled thin films comprised well-aligned, homogenous columns with similar height and diameter. To determine the average size of copper columns and the degree of uniformity in their alignment, the CGC films were studied using atomic force microscope (AFM). The measurements for a CGC film deposited at 100 W for 15 minutes are shown in Fig. 2c. The average grain size was 43.5 nm and the height of the columnar grains varied with sputtering time and power conditions. With longer sputtering times, the grains attained higher aspect ratios. Columnar grains even over 1 μm in height were grown in the film sputtered at 100 W for 60 minutes (Figure S1.a). The root mean square (RMS) roughness of the film was 0.829 nm, which indicates that the height of the columns was nearly constant.

Three different microstructures from various sputtering power conditions were observed using X-ray diffraction (XRD): amorphous, texture-controlled and polycrystalline structures (Fig. 2d). Amorphous copper grains formed at 20 W sputtering power. A (111) texture developed as the power reached 40 W, and the texture stabilized up to 100 W. Higher intensity diffraction patterns appeared at higher sputtering powers. The notable peak at (111) indicated that grains grew in a single orientation in the shape of columns. At 200 W and 300 W, additional intensified peaks at (200) and (220) appeared, suggesting the development of polycrystalline structures. Columnar grains in a selective orientation are a result of minimizing the crystallographically anisotropic free energy in the thin film system: surface energy, interfacial energy and strain energy are known to influence selective grain growth^19^. According to a previous study^20^, minimization of surface and interface energies are dominant in our experimental range of film thicknesses and sputtering conditions. In particular, with face-centered-cubic materials like copper, the (111) orientation has the minimum surface energy, leading to selective grain growth in this preferable orientation. When copper films are deposited with higher sputtering powers than 100 W, the minimization of strain energy governs the grain growth instead of surface and interfacial energy in the (100) and (110) orientations^19^,^21^. Therefore, our results are consistent with a general phenomenon in polycrystalline structures in copper films.

The deposition rates of CGC films are shown in Figure S1, with all thicknesses measured by AFM. The copper films were grown at a rate of 1 μm per hour at a sputtering power of 100 W. There was a linear dependence between deposition time and film thickness (Figure S1.a). In addition, for films deposited for 60 minutes, higher deposition rates were attained at higher sputtering powers, but the relationship was not linear (Figure S1.b).

**Figure 3:**
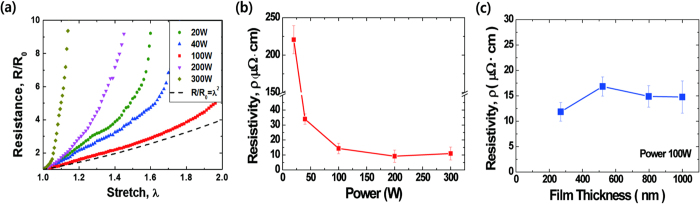
Experimental results—Resistance ratio of stretched copper films and resistivity curves as functions of sputtering power and film thickness. (**a**) Electrical resistances of CGC films as a function of applied strain. R_0_ and R denote the resistance of CGC films before and after uniaxial tension, respectively. The R/R_0_ of copper polycrystalline films (200 W and 300 W) surged up upon further elongation while that of the amorphous film (20 W) varied more gradually in comparison. While the other Cu films diverted significantly from the theoretical values at lower strains due to the formation of channel cracks, CGC films with (111)-oriented grains were more stretchable, maintaining their electrical conductivities at higher strain levels than any other type of copper films. At 100 W, the texture-controlled CGC film showed the most similar conductivity-strain behavior to the theoretical prediction for strains up to 100%. Further deformation was terminated by fracture of the PI substrate at 100% applied strain. (**b**) Resistivities of copper films before applying tension as a function of sputtering power. The resistivities tended to decline as power increased. At 20 W, the resistivity was higher by about an order of magnitude than in the other copper films. The resistivities of copper films deposited at above 100 W were around 15 μΩ · cm. (**c**) Measured resistivities of unstretched CGC films with various sputtering times were nearly identical, regardless of sputtering time or thickness of the CGC films. CGC films with different thickness values were deposited with different sputtering times at a constant power of 100 W (figure S1. a). Error bars show standard deviation; sample size n = 3.

The stretchability of the CGC films is shown in Fig. 3, determined by measuring the electrical resistance of the films during uniaxial tension. Mechanical straining and electrical measurements were performed on a custom-made stretcher made from anodized alumina for electrical insulation, as shown in Figure S2. The resistances of copper films deposited using different sputtering powers for 60 minutes are shown in Fig. 3a. In the case of the amorphous copper film grown at 20 W, the resistance at 40% strain quadrupled and rapidly increased tenfold at just below 60% applied strain. CGC films with controlled textures at 40 W and 100 W were the most stable, maintaining their electrical conductivities at higher strain levels than in any other cases. In particular, the CGC film deposited at 100 W showed the most similar conductivity-strain behavior to the theoretical prediction. The uniaxial elongation could not continue beyond 100% due to the rupture of the PI substrate, whereas the CGC film would have remained intact. The resistances in polycrystalline copper films deposited at 200 W and 300 W went up nearly tenfold under uniaxial elongation up to maximum strains of 40% and 10%, respectively. The relatively early discrepancy between the measured and the theoretical values in the amorphous and polycrystalline copper films was due to electrical pathway discontinuities originating from channel cracks and their propagations at small applied strain levels. More SEM images regarding on crack morphology under stretching are available in Figure S3 of Supporting information. The thicknesses of the films in Fig. 3a varied due to the different sputtering power conditions. The thickness effect on electrical resistivity was also explored in the Supporting information (Figure S4). While there was a slight dependence between film thickness and resistivity, it was weak relative to the effect of sputtering power.

Figure 3b shows the resistivities of copper films grown at different sputtering powers before tension. At a sputtering power of 20 W, where amorphous copper grains developed, the resistivity was an order of magnitude higher than those of the remaining cases. Grain sizes depending on sputtering powers were investigated in Figure S3. Larger grains were formed at higher sputtering powers. We suspect that the high electrical resistivity of copper films deposited at low sputtering powers may be due to their small grains and large amount of grain boundaries. At a sputtering power of 40 W, the resistivity dropped significantly to 34 μΩ · cm. Resistivities of copper films deposited above 100 W had almost uniform values around 15 μΩ · cm, indicating similar initial conductivity behavior between texture-controlled copper films and polycrystalline copper films. In Fig. 3c, we examined resistivity changes in CGC films during film deposition by measuring the resistivities at different film thicknesses. Deposited at a constant sputtering power of 100 W, the CGC film showed small variations in electrical resistivity, ranging from 10 to 15 μΩ · cm, implying that the resistivities of CGC films prior to straining remain constant during the deposition process. While reported resistivity of copper thin films on polyimide substrates is ranging from 3.8 to 17 μΩ · cm^22^,^23^. CGC films have slightly higher resistivity. High resistivity may be due to lots of grain boundaries in CGC films.

**Figure 4:**
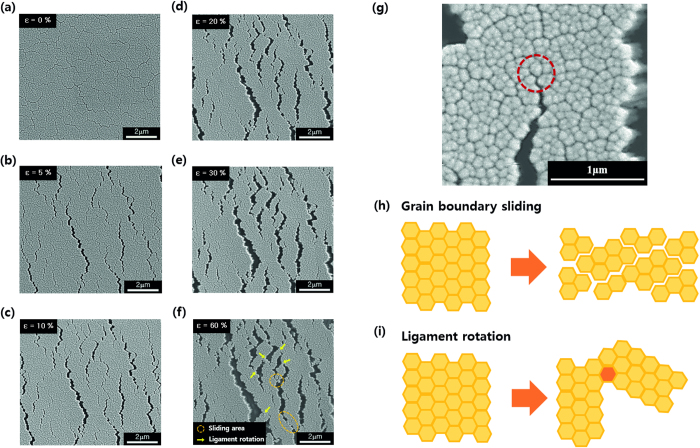
*In situ* SEM images and deformation mechanisms of stretched CGC films. (**a–f**) *In situ* SEM images of CGC film, formed at a RF sputtering power of 100 W, during uniaxial elongation up to 60%. (**a**) CGC film before applying strain. (**b**) With applied tension, initial cracks emerged under a strain of 5%. (**c**) The cracks propagated until 10% strain. After 10%, the cracks did not propagate further even though the CGC films were continuously stretched. This maintained a constant total density of cracks. (**d–f**) Instead of crack propagation, grains slippage and ligaments rotations occurred with increasing strain. The ligaments withstood strain up to 60%. Positions where sliding and ligament rotation occur are marked with circles and yellow arrows, respectively (**f**). A related movie is available in Supplementary Movie 1. (**g–i**) Deformation mechanisms of lengthened CGC films. (**g**) A short-range crack is trapped, and the extra load is easily shifted to other grains at small deformation levels, which leads to the development of ligaments. As the applied stress increases, two phenomena occur extensively: (**h**) sliding of grain boundaries and (**i**) rotation of ligaments. These grain movements compensate for the external load; thus, the ligaments can be sustained at larger deformations.

We captured *in situ* SEM images during uniaxial tension of CGC films deposited at 100 W. Strain proceeded up to 60%, with initial cracks generating at 5% strain. These cracks propagated with higher applied strain but were quickly followed by the activation of additional short cracks rather than the aggravation of existing cracks. When the strain reached 10%, the total density of cracks remained constant and further propagations were suppressed. According to the *in situ* SEM images, the ligaments endured applied stress up to 60% (Fig. 4d–f). A corresponding movie clip is available in Supplementary Movie 1. In Fig. 4g, an activated crack was trapped by other grains nearby, and the crack did not propagate with further elongation of the film. The localization of short-range cracks promoted the generation of other cracks at 10% strain through the transfer of load to neighboring grains. When larger strains were applied to the CGC film, grains tended to slide along grain boundaries (Fig. 4h), and some portions of the ligament structure rotated about a reference point (Fig. 4i). Crack trapping, grain boundary sliding and ligament rotations played a key role in maintaining electrical conduction beyond 100% applied strain.

## Conclusion

Columnar grown copper films on polyimide substrates were fabricated by controlling sputtering powers, and their electrical conductivities were measured during uniaxial tension. At a specific sputtering power condition, homogeneous columnar grains with uniform height and diameter and a selective texture of (111) orientation formed as a crystallographic product of minimizing the free energy of the system. The microstructural phenomena of crack trapping, grain boundary sliding and ligament rotation promoted high stretchability of the CGC films even beyond 100% applied strain, and their variation in electrical resistivity under strain approached the theoretically predicted behavior. In addition, with longer deposition times, columnar grains with higher aspect ratios were obtained, some of which were over 1 μm in height.

CGC films will be able to satisfy great demands in both research and industrial sectors for large-scale flexible electronics. The performance of these films can be optimized with the formation of high aspect ratio features modulated by sputtering powers and times. Stretchable interconnects with ligaments made from other metals deposited on other stretchable polymer substrates are also expected to be developed in further studies.

## Methods

### Fabrication procedure of Cu thin films

#### Preparation of polyimide (PI) substrate

Cu films were deposited on polyimide foils (Kapton® by DuPont) with dimensions of 5 mm × 40 mm × 125 μm. To improve adhesion, PI foils were sonicated in acetone for 10 minutes and then stored in a vacuum chamber for 2 hours to remove acetone remnants and absorbed water before deposition.

#### Copper deposition

Copper films were deposited by radio frequency (RF) sputter under various sputtering conditions, with powers ranging from 20 W to 300 W for different sputtering times, which produced copper films with thicknesses ranging from 200 nm to 1000 nm. The base pressure of the RF chamber was less than 10^−6^ torr. Ar was used as the working gas at a pressure of 2.5 × 10^−2^ torr. The radio frequency of the power supply was 13.56 MHz.

### Resistance measurement with uniaxial tensile tests on Cu films

The uniaxial tensile tests were performed with a custom-made micro tensile testing device (Figure S2). The screw driven stretcher was made of anodized alumina for electrical insulation. The initial length of each specimen was 5.6 mm. All tensile tests were conducted with a constant strain rate of 5 × 10^−4^ sec^−1^ at room temperature. The resistance of the films was measured by the 4-point probe method with a multimeter (DMM 2000/E, Keithley). A schematic of the entire experimental set-up is on Figure S2. (d). The microstructures of the copper films were observed in a scanning electron microscope (SEM) with a built-in focused ion beam (FIB) (NOVA200, FEI). *In situ* observations were also performed using the FIB-SEM system. The maximum applied strain was 100%, which was restricted by the failure of the PI substrates.

### AFM and XRD analysis

The average grain size, root mean square of surface roughness and film thickness were measured by atomic force microscopy (AFM, DI-3000, Digital Instrument) in contact mode in air at room temperature. Crystallographic textures of the copper films were examined by X-ray diffraction (XRD, M18XHF-SRA, MAC Science Co.) with Cu Kα radiation. The wavelength was 1.54056 Å and scanning speed was 10.0 degrees per minute. The voltage and current were 50.0 kV and 100.0 mA, respectively.

## Additional Information

**How to cite this article**: Sun, J.-Y. *et al.* Columnar grown copper films on polyimides strained beyond 100%. *Sci. Rep.* **5**, 13791; doi: 10.1038/srep13791 (2015).

## Supplementary Material

Supplementary Information

Supplementary Movie
