# Supplementary material for: Columnar grown copper films on polyimides strained beyond 100%
Source: Sci Rep. 2015 Sep 4;5:13791. doi: 10.1038/srep13791 (PMC4559799; doi:10.1038/srep13791)
Supplement: Supplementary Information [file srep13791-s1.pdf]

Supporting Information

Columnar grown copper films on polyimides strained beyond 100 %

Jeong-Yun Sun*, Hae-Ryung Lee, Kyu Hwan Oh*

Figure S1. Film thickness of copper films as a function of sputtering conditions - time and power

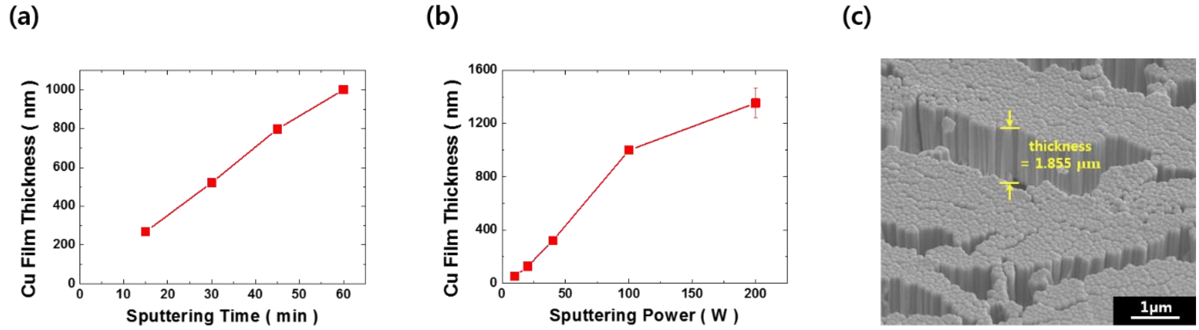

The thickness of the copper films, which scaled directly with the height of the columnar grains, was measured as functions of sputtering time (**a**) and power (**b**) by AFM. **(a)** The thickness values of copper films deposited at 100 W increased linearly with time. Columnar copper grains were grown to over 1 μm as the sputtering time was extended to 60 minutes. **(b)** The thickness of the copper films sputtered for 60 minutes also increased as higher powers were applied. **(c)** A SEM image of CGC columnar grown copper grains with thickness of 1.855 μm. The sample was deposited at sputtering power of 100 W for 60 minutes. The picture was taken with tilted angle of 52 ° after the film was applied 98.25 % strain.

Figure S2. A custom-made screw-driven tensile testing device and an experimental set-up for measuring electrical resistances in the strained copper films

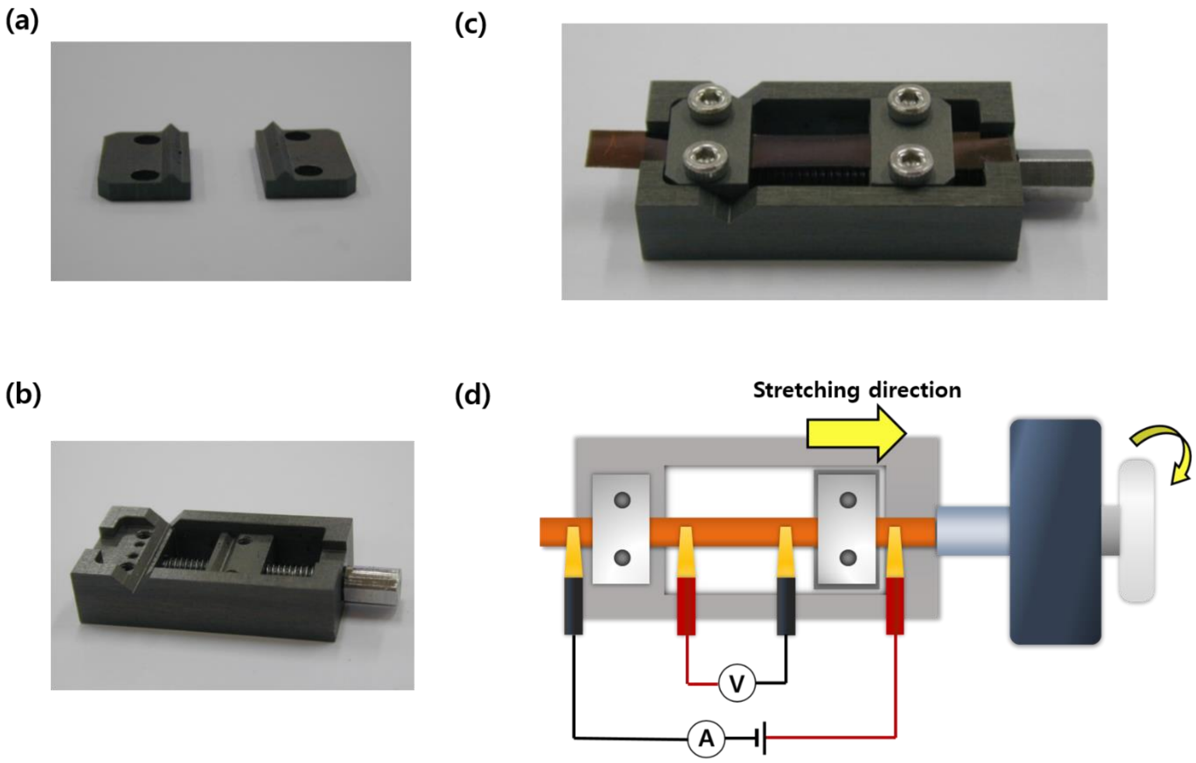

A custom-made experimental setup was prepared to measure the electrical resistances in the copper films. **(a)** and **(b)** represent pieces of the hand-made tensile testing device – two grips and a body, respectively. Each grip had a hinge, with grooves that fit into the body and two holes for bolts. The body contained a mobile stage, which could move horizontally inside the body as a screw was tightened or loosened by the knob. The body and grips were made of alumina, whose top layers were anodized for electrical insulation. For measuring the resistance of the strained copper films on PI substrates, the films were laid in the body and covered by the grips; one grip remained fixed while the other moved to perform tension **(c)**. **(d)** The strain was controlled by turning the knob at a constant rate of 0.05%/sec. Electrical resistivity of the stretched Cu film was measured by 4-point probe method.

Figure S3. SEM images of copper grains deposited at different RF sputtering power conditions

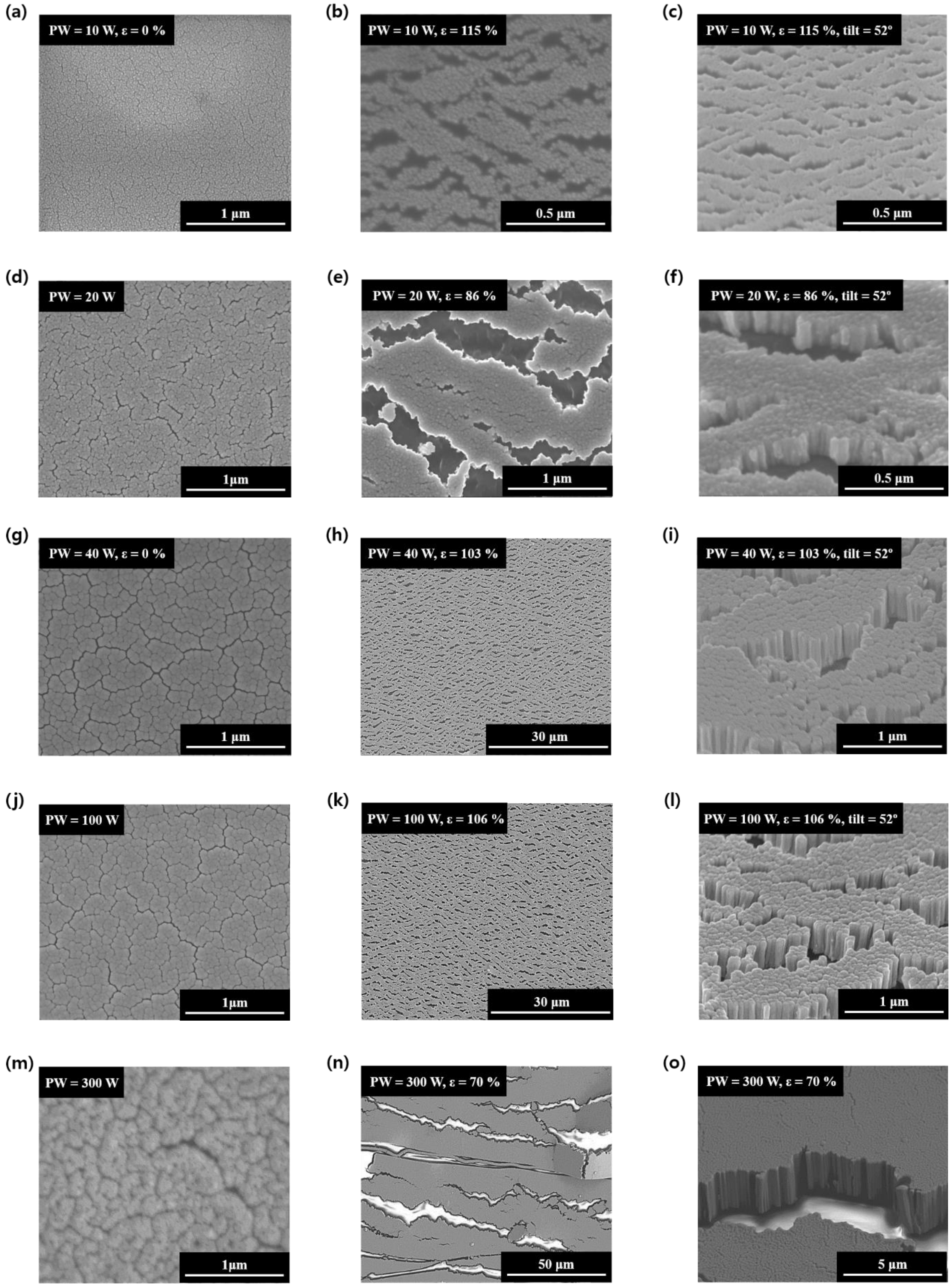

SEM images of copper films developed under various sputtering power conditions; **(a)-(c)** 10 W, **(d)-(f)** 20 W, **(g)-(i)** 40 W, **(j)-(l)** 100 W and **(m)-(o)** 300 W. The images on the first column are copper films before stretching. Larger grains were gained as sputtering powers went up. The second and the third columns have copper films after stretching; the middle line is top view of the stretched film and the remnant is tilted view of 52°. In case of stretched copper film which was developed at RF power of 300 W, morphology of crack propagation showed remarkable distinction from other cases **(n)**. All copper films were grown for 60 minutes except for the films deposited at 100 W, whose sputtering time was 15 minutes.

Figure S4 . Relative resistance of strained CGC films with different film thickness

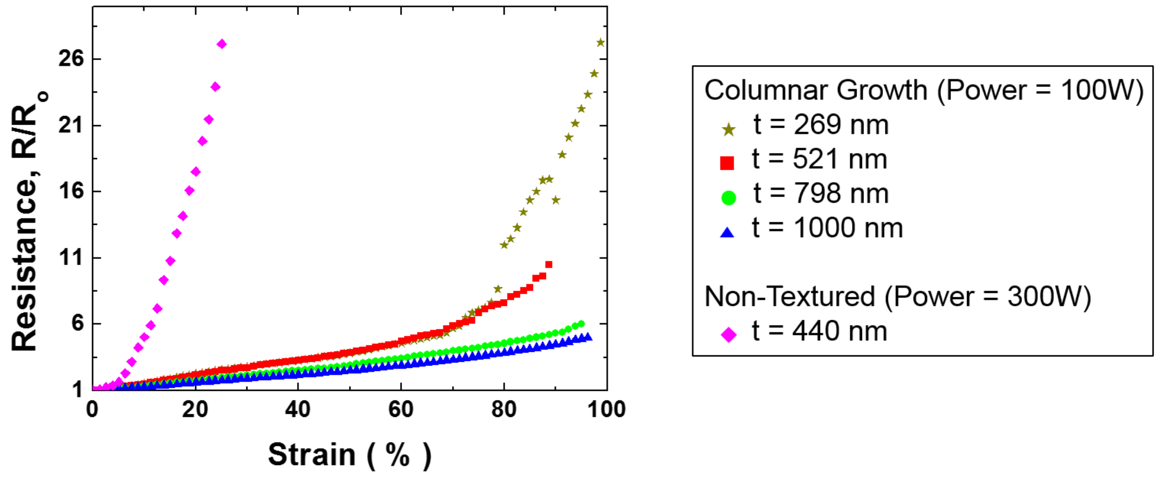

Relative electrical resistance of strained CGC films with different thickness values were explored by 4-point probe method. There was a small dependence between resistance and film thickness under the tensile test.

Supplementary Movie 1.

A columnar grown copper film (CGC film) deposited at a RF sputtering power of 100 W for 60 minutes was elongated in the horizontal direction up to 60 %, and the microstructural changes were observed by SEM. Initial cracks appeared at 5 % applied strain. Upon further straining, cracks remained trapped instead of propagating. Grain boundary sliding and ligament rotation accompanied the trapping of cracks, resulting in a ligament structure in the CGC film that would sustain electrical conduction.
