# Supplementary figures and images for: Columnar grown copper films on polyimides strained beyond 100%
Source: Sci Rep. 2015 Sep 4;5:13791. doi: 10.1038/srep13791 (PMC4559799; doi:10.1038/srep13791)

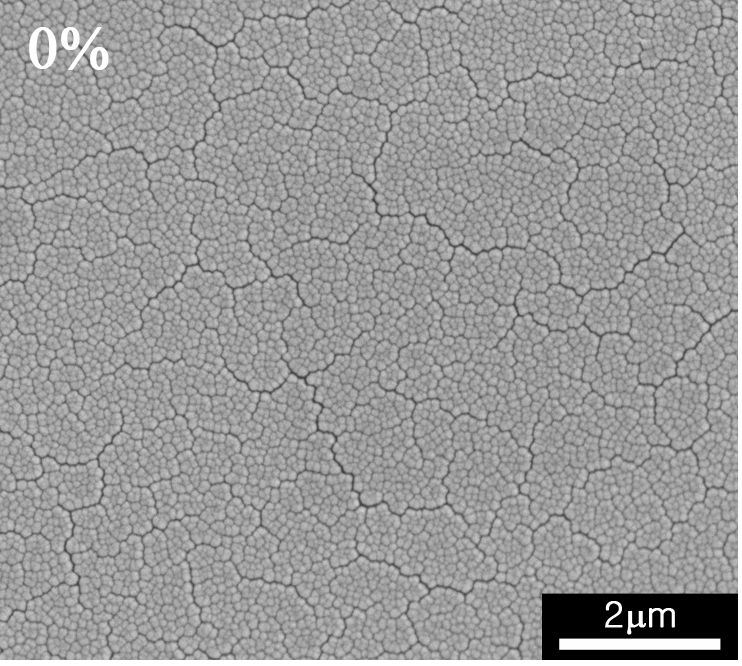

Supplement: Supplementary Movie [file srep13791-s2.gif]
